# Difference in sulfur regulation mechanism between tube-dwelling and free-moving polychaetes sympatrically inhabiting deep-sea hydrothermal chimneys

**DOI:** 10.1186/s40851-023-00218-5

**Published:** 2023-10-04

**Authors:** Tomoko Koito, Yusuke Ito, Akihiko Suzuki, Akihiro Tame, Tetsuro Ikuta, Miwa Suzuki, Satoshi Mitsunobu, Makoto Sugimura, Koji Inoue

**Affiliations:** 1 Department of Marine Science and Resources, Nihon University, 1866 Kameino, Fujisawa, Kanagawa 252-0880 Japan; 2 Present Address: National Institute for Environmental Studies, 16-2 Onogawa, Tsukuba, Ibaraki 305-8506 Japan; 3 Marine Works Japan, Ltd., 3-54-1 Oppamahigashi, Yokosuka, Kanagawa 237-0063 Japan; 4 Japan Agency for Marine-Earth Science and Technology (JAMSTEC), 2-15 Natsushima, Yokosuka, Kanagawa 237-0061 Japan; 5 Department of Science and Technology for Biological Resources and Environment, Ehime University, 3-5-7 Tarumi, Matsuyama, Ehime 790-8566 Japan; 6 Enoshima Aquarium, 2-19-1 Katase, Fujisawa, Kanagawa 251-0035 Japan; 7 Atmosphere and Ocean Research Institute, The University of Tokyo, 5-1-5, Kashiwanoha, Kashiwa-Shi, Chiba 277-8564 Japan

**Keywords:** Spherocrystal, Sulfur regulation, Hydrothermal vent, *Paralvinella* spp., Polynoidae. gen. sp

## Abstract

The environment around deep sea hydrothermal vents is characterized by an abundance of sulfur compounds, including toxic hydrogen sulfide. However, numerous communities of various invertebrates are found in it. It is suggested that invertebrates in the vicinity of hydrothermal vents detoxify sulfur compounds by biosynthesis of taurine-related compounds in the body. On the other hand, the vent endemic polychaete *Alvinella pompejana* has spherocrystals composed of sulfur and other metals in its digestive tract. It was considered that the spherocrystals contribute to the regulation of sulfur in body fluids. *Paralvinella* spp. and Polynoidae. gen. sp. live sympatrically and in areas most affected by vent fluid. In this study, we focused on the digestive tract of *Paralvinella* spp. and Polynoidae. gen. sp. to examine whether they have spherocrystals. We also investigated the possible involvement of bacteria in the digestive tract in spherulization. Examination with a scanning electron microscope (SEM) equipped with Energy Disperse X-ray Spectroscopy (EDS) detected spherocrystals containing sulfur and iron in the digestive tract of *Paralvinella* spp. In contrast, such spherocrystals were not observed in that of Polynoidae. gen. sp. although sulfur is detected there by inductively coupled plasma-optical emission spectrometry (ICP-OES). Meta-16S rRNA analysis indicated that the floras of the digestive tracts of the two species were very similar, suggesting that enteric bacteria are not responsible for spherocrystal formation. Analysis of taurine-related compounds indicated that the digestive tissues of Polynoidae. gen. sp. contain a higher amount of hypotaurine and thiotaurine than those of *Paralvinella* spp. Therefore, the two sympatric polychaetes use different strategies for controlling sulfur, i.e., *Paralvinella* spp. forms spherocrystals containing elemental sulfur and iron in the digestive tract, but Polynoidae. gen. sp. accumulates taurine-related compounds instead of spherocrystals. Such differences may be related to differences in their lifestyles, i.e., burrow-dweller or free-moving, or may have been acquired phylogenetically in the evolutionary process.

**Supplementary Information:**

The online version contains supplementary material available at 10.1186/s40851-023-00218-5.

## Background

Throughout the marine ecologies on earth, unique ecosystems of invertebrates have been discovered around deep-sea hydrothermal vents [1–3]. Such ecosystems are nutritionally supported by “chemosynthetic bacteria” that produce organic matter using the chemical components included in vent fluids [4, 5]. Hydrogen sulfide is one of the major components used for chemosynthesis, and the bacteria conducting this reaction are called sulfur-oxidizing bacteria [6, 7].

Vent-specific invertebrates usually inhabit the positions where chemosynthesis products are available, i.e., near the vents [8]. The closer the position is to a vent, the richer in nutrients. However, such positions are also exposed to hydrothermal fluid containing high levels of hydrogen sulfide, which can permeate the body walls of invertebrates [9] and thereby hamper the organisms. For example, exposure to high concentrations of sulfide causes mitochondrial depolarization [10]. Sulfides also cause oxidative damage to DNA and RNA, inducing mutations such as G-T transversions [11]. Therefore, vent-specific invertebrates, especially those inhabiting positions close to vents, must evolve mechanisms to adapt to the toxicity of hydrogen sulfide.

Polychaetes are among the major occupants of hydrothermal vent ecosystems [12, 13]. Some of the polychaete species are known to prefer positions directly exposed to vent fluids. For example, at the hydrothermal vents in Myojin Knoll Caldera in Izu-Ogasawara Area of the North-Western Pacific Ocean, the polychaetes *Paralvinella hessleri* and Polynoidae are observed to occupy positions near the upper parts of the chimneys of the vents [14–18]. Therefore, such vent-specific polychaetes are thought to have mechanisms to cope with the toxicity of hydrogen sulfide. In siboglinid tubeworms and vesicomyid clams, specific components that bond to hydrogen sulfide and circulate it in a nontoxic state have been discovered [19, 20]. However, such components have been reported only from limited species. Another possible mechanism to cope with the toxicity of hydrogen sulfide is the use of a taurine-related compound, hypotaurine, which binds to sulfide ion and becomes non-toxic thiotaurine [21–23]. In the bivalves and siboglinids, positive relationships between the amount of thiotaurine and the concentration of hydrogen sulfide in the habitat have been suggested [24, 25]. In addition, experimental exposure to sulfide is also reported to increase thiotaurine levels in bivalves, siboglinids, and paralvinellid worms [23, 26].

In a previous study, we quantified the levels of taurine-related compounds (taurine, thiotaurine, and hypotaurine) in two above-mentioned deep-sea polychaete species, *P. hessleri* and Polynoidae. gen. sp. collected from the Myojin Knoll [16]. These two species live sympatrically where they are most exposed to vent fluid. The two species differ greatly in morphology and are easily distinguished. In addition, any active chimney in the Myojin Knoll area has both species attached to it, which has the advantage of eliminating the need for researchers to spend long hours searching for the target organism on the seafloor. Our results indicated that abundance of hypotaurine and thiotaurine in *P. hessleri* was significantly lower than that in the Polynoidae. gen. sp. This suggests that degree of dependence on the hypotaurine/thiotaurine system is different between the two sympatric polychaetes, and *P. hessleri* may have another mechanism to adapt to sulfide-rich environments [16].

In the gastric epithelial cells of *Alvinella pompejana*, which is endemic to hydrothermal vents and belongs to the same family as *P. hessleri*, spherocrystals containing metallic elements such as iron and elemental sulfur exist [27]. Although the formation process of the spherocrystals is unknown, they are assumed to be involved in the regulation of iron and sulfur levels in the blood and digestive fluid [27]. In this study, we hypothesized that *Paralvinella* spp., containing a small amount of hypotaurine and thiotaurine, regulates internal sulfur by spherulizing it. We observed the digestive tract of *Paralvinella* spp. and Polynoidae. gen. sp. isolated from the same chimney piece using SEM–EDS. As a result, S- and Fe-containing spherocrystals were detected only from *Paralvinella* spp. In addition, contents of total sulfur and iron in the digestive tract and other parts were also quantified by ICP-OES. Subsequently, bacterial flora in the digestive tract was also analyzed by partial 16S-rRNA amplicon sequencing to examine the contribution of bacteria to the spherocrystal formation. We also analyzed the contents of taurine-related compounds in the digestive tract. Based on the results, we discuss the differences in sulfur regulation of the two sympatric vent-specific polychaetes.

## Materials and methods

### Sample collection

*Paralvinella* spp. and Polynoidae. gen. sp. were collected from the chimney in Myojin Knoll Caldera, Izu-Ogasawara Arc. About 30 cm pieces of the chimneys were collected at 32°06.2202´N/139°52.1497´E (depth; 1,223 m) and 32°06.2225´N/139°52.1439´E (depth; 1,223 m) using the arm of the remotely operated vehicle (ROV) *Hyper-Dolphin*, operated by the research vessel (R/V) *Shinsei Maru* during KS-18–3 (April 3–9, 2018) and KS-20–1 (January 7–11, 2020) cruises. The chimney piece was kept in an insulated box until recovery of the ROV. Immediately after recovery, Polynoidae. gen. sp. on the surface of the chimney was collected, and *Paralvinella* spp. bodies were removed from their tube’s chimney using forceps. For SEM observation, Polynoidae. gen. sp. and *Paralvinella* spp. were fixed with 2.5% glutaraldehyde in filtered seawater and stored at 4 °C. For analysis of elemental and taurine-related compounds, the samples were immediately frozen using liquid nitrogen. For bacterial flora analysis, the samples were fixed in 99.5% ethanol. After the cruise, frozen samples were dissected with a disposable scalpel on a plastic dish placed on ice using a tabletop inverter loupe. They were divided into branchiae, digestive tract, and remaining parts (hereafter called ‘body wall’). The digestive tract was isolated by opening the abdominal cavity. As the esophageal gland and stomach of *Paralvinella* spp. were indistinct at the magnification of the loupe, the tubular part from the mouth was used as the digestive tract. Polynoidae. gen. sp. had a clear esophageal gland, but the other tissues were indistinct, so the esophageal gland and the tubular portion that followed it were designated as the digestive tract. See additional files for details on dissection (Additional files 1 and 2). In addition, the branchia had a small amount of tissue, and in order to avoid a quantitative shortage in various analyses, tissue other than the digestive tract containing the branchiae was collected as the body wall.

### SEM observation and EDS analysis

The samples fixed with 2.5% glutaraldehyde in filtered seawater were washed with filtered artificial seawater, then dehydrated using a series of graded ethanol (30, 50, 70, 90, and 100%), and embedded in Technovit 8100 resin (Kulzer) at 4 °C. Semi-thin Sects. (2 μm thickness) were cut using a glass knife mounted on an Ultracut S ultra-microtome (Leica Microsystems), collected on glass slides, and coated with osmium (10 nm layer thickness) using an OPC-80 osmium coater (Filgen). The sections were observed and analyzed using a Quanta 450 FEG field-emission SEM with backscattered electron detector and EDS (FEI) operating at 5 and 15 kV.

### Elemental analysis

The branchiae, digestive tract, and body wall excised from the frozen samples were placed in an oven at 85 °C and dried for 24 h. After the dry weight was measured, the sample was dissolved for over 6 h with the addition of 1 M hydrochloric acid. The dissolved sample was centrifuged at 8163 g for 5 min. The supernatants were diluted to 3 mL by 0.1 M HCl. Total sulfur concentration was determined by ICP-OES (Varian, 730-ES). For quality control purposes, standard trace grade solutions containing S and Fe in sample range concentrations were prepared and analyzed. All standard solutions were quantified within the range of ± 5% of the reported values.

### Bacterial flora analysis

The digestive tracts were isolated from one each 99.5% ethanol-fixed individual of *Paralvinella* spp. and Polynoidae. gen. sp. using a disposable scalpel. Isolated samples were put into 1 ml of phosphate-buffered saline (PBS) and centrifuged at 10,000 g for 1 min, and the supernatant was discarded. This process was repeated three times. After final washing, pellets were resuspended in 700 μL of buffer RLT (Qiagen, Hilden, Germany)-99% 2-mercaptoethanol solution (100:1 v/v). Then, samples were homogenized with 0.5 mm diameter glass beads using a bead-based homogenizer. The homogenates were shaken after the addition of 700 μL of phenol–chloroform-isoamyl alcohol (PCI; 25:24:1 v/v/v) solution and centrifuged at 16,000 g for 3 min. The upper layer was again extracted with PCI. Finally, 300 μL of the upper layer was collected, to which 30 μL of 3 M sodium acetate, 3 μL of Ethachinmate (Nippon Gene, Tokyo, Japan), and 750 μL of 99% ethanol were added, and centrifuged at 20,000 g for 3 min. After removing the supernatant, the DNA was dissolved in 50 μL of Buffer AE (Qiagen). The concentration of extracted DNA was measured using a NanoDrop Lite spectrophotometer. Bacterial 16S rRNA V3-V4 region was amplified by PCR using the bacterial universal primers (Bakt_341F: 5′-CCTACGGGNGGCWGCAG-3′ and Bakt_805R: 5′-GACTACHVGGGTATCTAATCC-3′ [28]). The PCR reaction and preparation of amplicon pool were conducted following the methods of Suzuki et al. [29]. The resultant library was sequenced onto a MiSeq flowcell for the 250 bp paired-end sequencing protocol. Data analyses were conducted using CLC Genomics Workbench (CLC Bio, Aarhus, Denmark). From the raw sequence data, index and adaptor sequences were trimmed, and low quality (< Quality Score 30) and short length (< 400 bp) reads were removed. The homology search with the basic local alignment search tool (BLAST) for bacterial 16S rRNA at > 98% identity level was performed using Metagenome@KIN software (World Fusion, Tokyo, Japan).

### Taurine-related compounds analysis

Taurine, hypotaurine, and thiotaurine were extracted from the digestive tract and body wall of frozen samples. Both tissues were weighed and 2–3 volumes of chilled 80% ethanol and 2 μL of the internal standard, and norlerucine (50 nmμL ^−1^ in water) were added. Samples were homogenized using a bead-based homogenizer. The homogenate was centrifuged at 15,000 g for 10 min, and 20 μL of the supernatant was dried in a vacuum centrifuge. After evaporation, the pellets were dissolved in 20 μL of ammonia (28%)-methanol solution (7:3 v/v) and again dried in a vacuum centrifuge. Derivatization of the sample was performed by dissolving it in 20 μL of methanol-ammonia-phenyl isothiocyanate solution (7:2:1, v/v), and adding 500 μL of Pico-Tag solution (Waters Corporation, Milford, MA, USA) [16]. Amino Acid Mixed Standard H (Wako Pure Chemicals, Osaka, Japan), hypotaurine, β-alanine, taurine, thiotaurine, and norleuchine were used as chemical standards by dissolving them in 0.1 N HCl. The standards were also dried and derivatized as described above. The samples and standards were then filtered through 0.45-μm filters (Millipore, Billerica, Massachusetts, USA) and PITC-labeled taurine-related compounds and other amino acids were detected by reversed-phase high-performance liquid chromatography using a gradient program described by Nagasaki et al. [30].

### Statistical analysis

The statistical significance of differences among the samples was evaluated using univariate analysis of variance (ANOVA) with a Scheffe’s F test.

## Results

### SEM observation

#### *Paralvinella* spp.

**Fig. 1:**
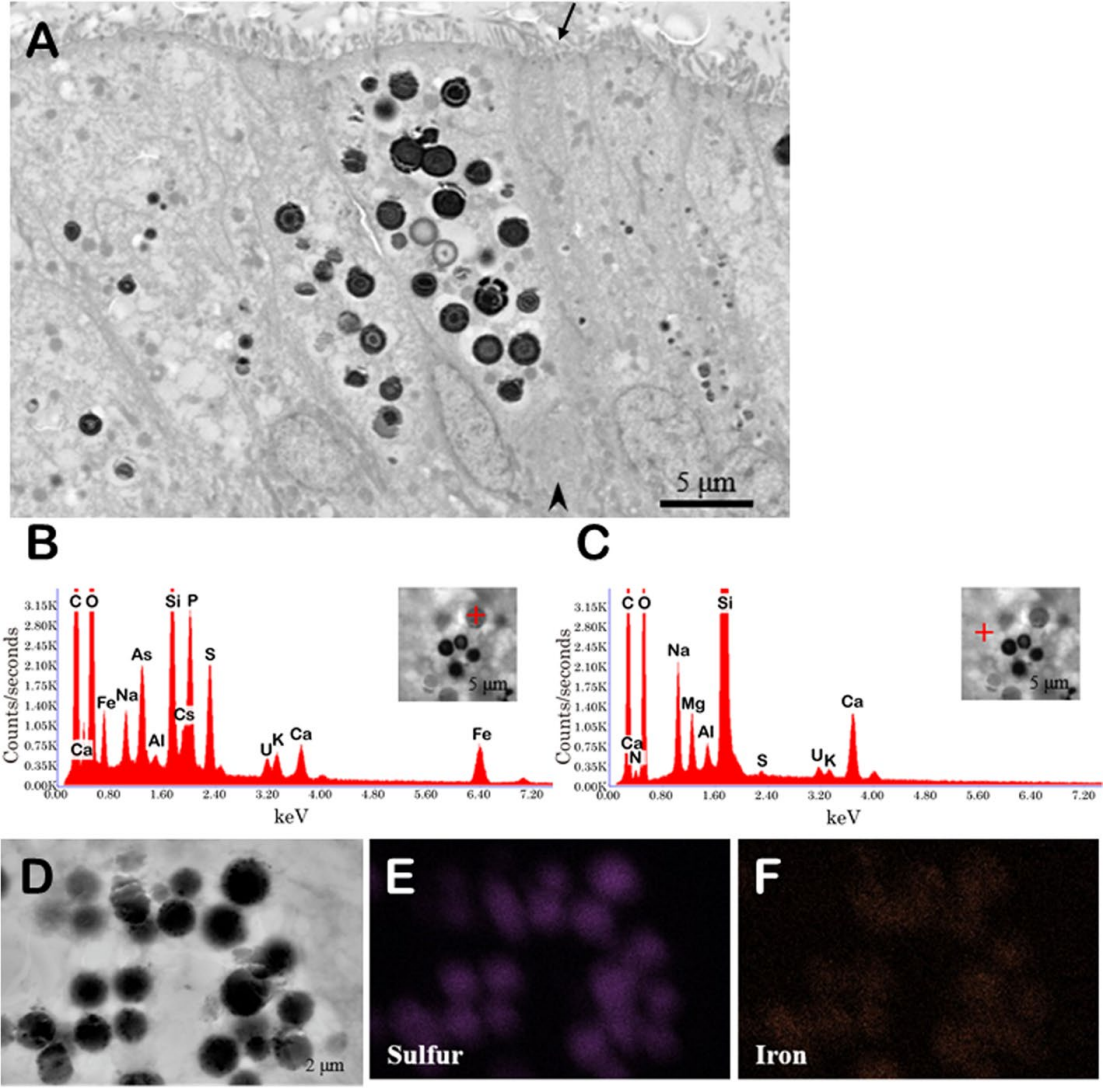
Intestinal epithelial cells of *Paralvinella* spp. and its EDS mapping*.* **A**: SEM micrograph of intestinal epithelial cells. Arrow and arrowhead indicate apical and basal side, respectively. **B**: EDS spectrum of a spherocrystal. **C**: EDS spectrum of non-spherocrystal position. **D**: SEM image of spherocrystals. **E**, **F**: EDS sulfur and iron mapping of the region shown in **D**

SEM images of *Paralvinella* spp. sections revealed the presence of numerous electron-dense spherocrystals in the gut epithelial cells. The spherocrystals were distributed around the digestive tract and there were several dozen grains per cell in the visual field. These all had a nearly spherical shape, were approximately 1 μm in size, and ranged from apical to near basal (Fig. 1A). Qualitative analysis with EDS detected As, P, S, K, and Fe elements in the spherocrystals. C, O, Na, Mg, Al, and Ca elements were also detected in the background without spherocrystals (Fig. 1B, C). S and Fe elements were localized in the spherocrystals as revealed by EDS mapping (Fig. 1D–F).

#### Polynoidae. gen. sp.

**Fig. 2:**
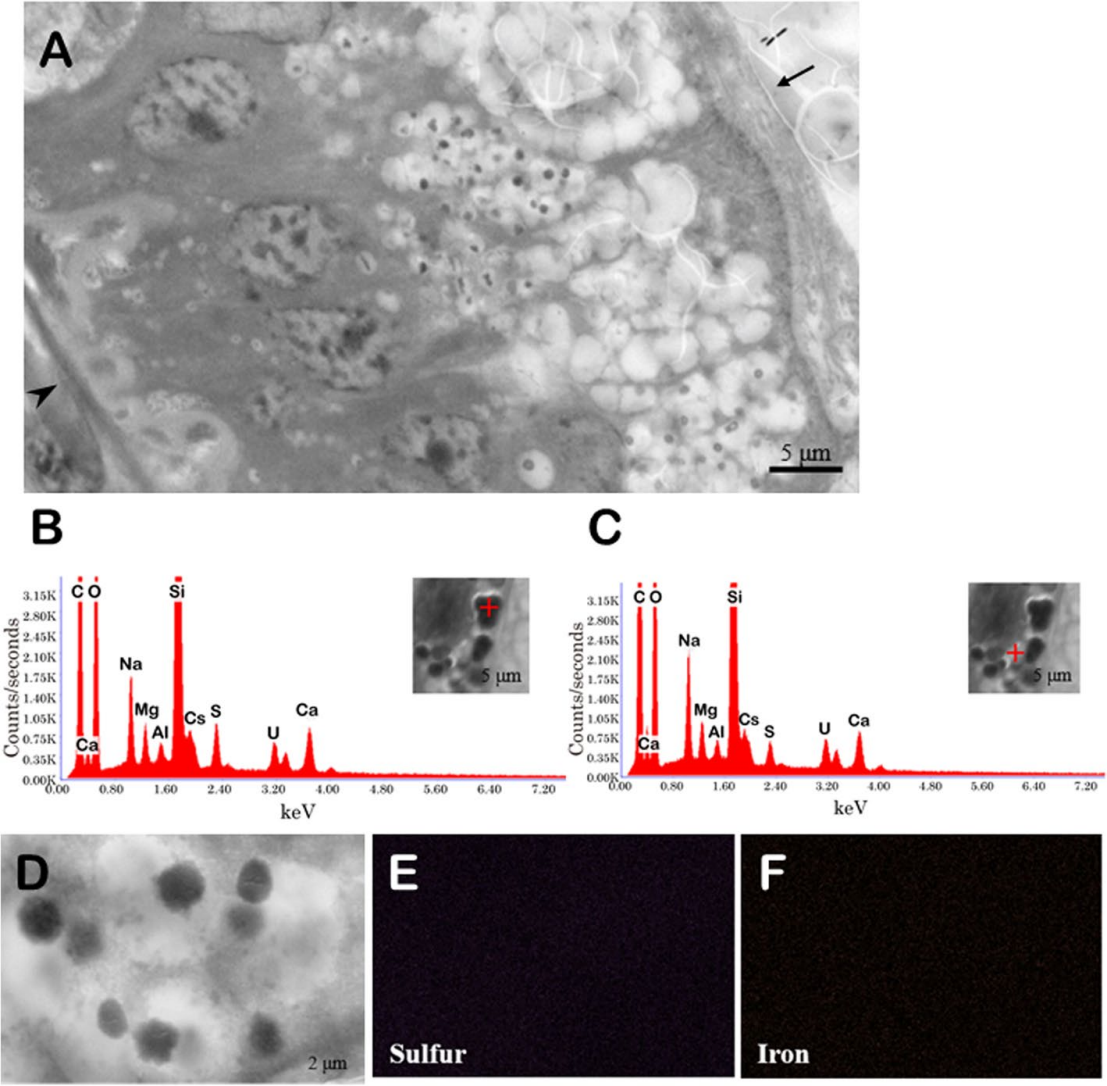
Intestinal epithelial cells of Polynoidae. gen. sp. and its EDS mapping*.* **A**: SEM micrograph of intestinal epithelial cells. Arrow and arrowhead indicate apical and basal side, respectively. **B**: EDS spectrum of a granule. **C**: EDS spectrum of non-granule position. **D**: SEM image of granules. **E**, **F**: EDS sulfur and iron mapping of the region shown in **D**

SEM showed the presence of electron-dense granules in the digestive tract of the Polynoidae. gen. sp. (Fig. 2A). The granule size was more variable than that of *Paralvinella* spp. They were also close to ellipsoids in shape, with a mixture of high and low numbers per cell. In the qualitative analysis by EDS, C, O, Na, Mg, Al, Ca, S, and K were detected in electron-dense granules as well as in the background without granules (Fig. 2B, C). S and Fe were not localized in the granules as they were in *Paralvinella* spp. (Fig. 2D–F).

### Elemental analysis

In both species, S was detected in the digestive tract although it was more abundant in the body wall of both species: mean values were 8.4 and 7.9 times higher than that in the digestive tract, for *Paralvinella* spp. and Polynoidae. gen. sp., respectively (Table 1). The branchiae of the *Paralvinella* spp.were also rich in S. Fe was detected in the digestive tract and the body wall of *Paralvinella* spp. although that in the latter was at low levels. Fe was below the detection limit in the branchiae. It was detected in the body wall of Polynoidae. gen. sp. but it was below the detection limit in the digestive tract. However, differences among tissues or between species were not statistically significant because of large individual differences for both elements.

**Table 1:**
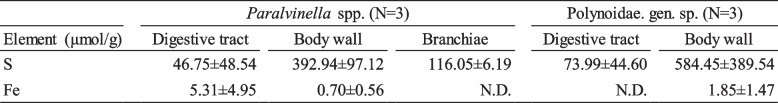
The mean levels (± SE) of sulfur and iron element in the tissues of *Paralvinella* spp. and Polynoidae. gen. sp.

### Bacterial flora

Bacterial floras in the digestive tract of the two polychaetes were analyzed by partial 16S rRNA amplicon sequencing. At the phylum level, both species were dominated by Proteobacteria, which accounted for about 95% and 82% for *Paralvinella* spp. and Polynoidae. gen. sp., respectively (Table 2). For other phyla, Bacteoidetes and Actinobacteria occupied about 0.1% each in *Paralvinella* spp., and Firmicutes accounted for 0.23% in Polynoidae. gen. sp. (Table 2). Other phyla were hardly detected. At the bacterial species level, gut flora of *Paralvinella* spp. and Polynoidae. gen. sp. were similar (Table 3). The composition of abundant species was very similar in *Paralvinella* spp. and Polynoidae. gen. sp. *Varivorax boronicumulans* was the most abundant species, and accounted for about 20% in both species (Table 3), and *V. paradoxus*, *V. guangxiensis*, and *Xenophilus arseniciresistens* followed it. The bacterial species list with the number of read counts in the digestive tract of *Paralvinella* spp. and Polynoidae. gen. sp. is shown in Additional file 3.

**Table 2:**
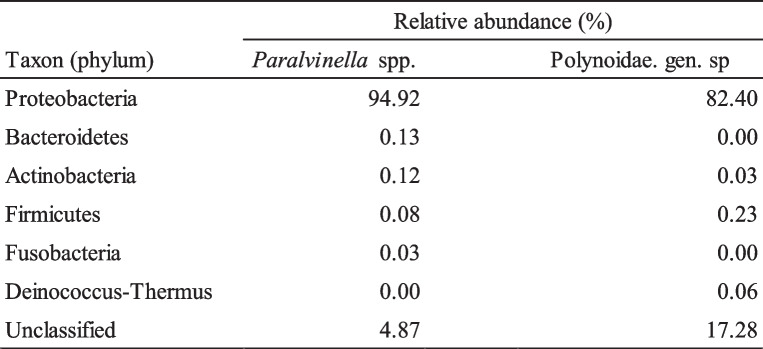
Relative abundance of the bacterial phyla in the digestive tracts of *Paralvinella* spp. and Polynoidae. gen. sp.

**Table 3:**
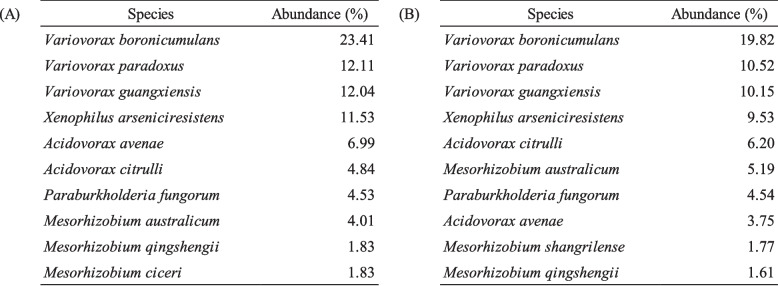
Top 10 bacterial species and its abundance (%) detected from the digestive tract of *Paralvinella* spp. and Polynoidae. gen. sp. (A) *Paralvinella* spp., (B) Polynoidae. gen. sp.

### Taurine-related compounds

In *Paralvinella* spp., concentrations of the three taurine-related compounds, taurine, hypotaurine, and thiotaurine were lower in the digestive tract than in the body wall and branchiae (Table 4). Additionally, taurine was the most abundant among the three compounds in all the three body parts. In contrast, the tissues of Polynoidae. gen. sp. contained high levels of hypotaurine and thiotaurine compared with *Paralvinella* spp. Hypotaurine + thiotaurine/hypotaurine + thiotaurine + taurine was calculated for dependency comparison to the hypotaurine/thiotaurine system. The results showed that Polynoidae. gen. sp. had a higher proportion in all tissues than *Paralvinella* spp. (Table 4). The body wall of Polynoidae. gen. sp. exhibited the highest mean value, 0.78, and the branchiae of *Paralvinella* spp. showed the lowest mean value, 0.09.

**Table 4:**
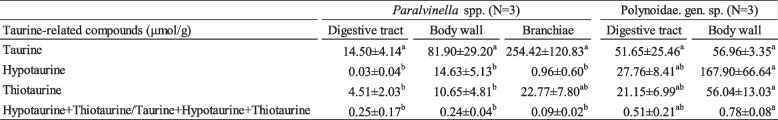
The mean levels (± SE) of taurine-related compounds in the tissues of *Paralvinella* spp. and Polynoidae. gen. sp. Different superscript letters show significant differences (*p* < 0.01) in the same row

## Discussion

In this study, spherocrystals composed of metals and S were observed in the digestive tract of *Paralvinella* spp. (Fig. 1). The spherocrystals of *Paralvinella* spp. were very similar in shape to those observed in the *Alvinella* intestine [27]. In addition, S and Fe were detected by elemental analysis using ICP-OES in the digestive tract of the *Paralvinella* spp., which was consistent with EDS results (Fig. 1B). Electron-dense granules were also observed in the digestive tract of Polynoidae. gen. sp., but these granules contained less sulfur and metals, localized at the apical side, and presumably contained mucus (Fig. 2A). These granules looked similar to those reported in secretory or digestive cells of the intestinal tract of the shallow-water polychaete *Eulalia viridis* [31]. Thus, the granules in the cells of the digestive tract of Polynoidae. gen. sp. were considered irrelevant to sulfide regulation, digestion, and absorption. The elemental analysis by ICP-OES detected more S in the digestive tract of Polynoidae. gen. sp. than in that of *Paralvinella* spp. (Table 1). Elemental mapping by EDS showed that S was scattered in the visual field and did not overlap the localization of the particles in Polynoidae. gen. sp. (Fig. 2E). Thus, the digestive tracts of both species contain S, but only *Paralvinella* spp. forms spherocrystals, and Polynoidae. gen. sp. has S in another form without forming spherocrystals, for example, in the form of sulfur-containing amino acids or proteins.

The bacterial floras of the digestive tract of the two polychaete species were found to be very similar. In both polychaete species, *Varivorax*, *Xenophilus*, and *Acidvorax*, belonging to the family Comamonadaceae, occupied the upper rank in the flora analysis (Tables 3 and 4). They are known to be resistant to metals and have sulfide metabolizing systems [32–34]. It is reasonable to find them in sulfur- and metal-rich digestive tracts of the vent-endemic polychaete. The involvement of symbiotic sulfur-oxidizing bacteria in the formation of sulfur crystals has been suggested in the siboglinids [35, 36]. The similarity of the bacterial flora of the two species suggests that the formation of spherocrystals does not involve bacteria in the digestive tract. Thus, spherocrystals are likely to be formed by the polychaete’s own metabolism in the digestive tract of *Paralvinella* spp. and not by bacterial metabolism.

The functions of the spherocrystals are unknown at present. A possible role of the spherocrystals of *Paralvinella* spp. is to function as the media for sulfur and/or sulfide storage. In addition, the spherocrystals may also store other metals and minerals. In this study, Fe, P, Ca, and As were detected in the spherocrystals (Fig. 1B). In *Alvinella pompejana*, *A. caudata*, and *Paralvinella grasslei,* trace elements such as copper, zinc, cadmium, and arsenic are detected in their anterior parts, branchial tentacles, and digestive tract, mainly in insoluble forms [37, 38]. Thus, storage of trace metals in insoluble forms may be a characteristic of tube-dwelling alvinellid worms. Interestingly, metallic spherocrystals in the intestinal tract have also been detected in the polychaetes *Owenia fusiformis* and *Hediste diversicolor*, which are tube- or burrow-dwelling in shallow waters [39, 40].

At least in this study, no spherocrystals like those in *Paralvinella* spp. were found in the digestive tract of Polynoidae. gen. sp., but taurine-related compounds were detected at high levels (Table 4). The ratio of hypotaurine + thiotaurine/hypotaurine + thiotaurine + taurine is a high sulfur regulatory mechanism. In addition, the body wall contained larger amounts of hypotaurine and thiotaurine than the digestive tract (Table 4). As Polynoidae. gen. sp. does not have a tube and walks on the surface of vent chimneys using parapodia, the body surface is constantly exposed to hydrogen sulfide. Therefore, for this species, the hypotaurine/thiotaurine system is likely to be a major mechanism of sulfur metabolism throughout its body. Hypotaurine is also known to contribute to cellular protection against oxidative stress in many organisms and also to cellular osmoregulation [41–43]. Indeed, deep-sea Polynoidae exhibits repellent behavior at high temperatures, i.e., at high hydrogen sulfide concentrations [44, 45]. In other words, if they encounter high levels of hydrogen sulfide instantaneously while traveling, the large amounts of thiotaurine and hypotaurine that accumulate may contribute to tissue protection. Even in *P. sulfincola*, the branchiae, which are directly exposed to vent fluid, contained a certain amount of thiotaurine [23]. Therefore, dependence on the hypotaurine/thiotaurine system is thought to be related to the lifestyle of each species.

*Paralvinella* spp. and Polynoidae. gen. sp. belong to taxonomically distinct orders. In gastropods, the elements that form spherocrystals vary from species to species and are broadly divided into four groups, with food and taxonomic position determining which group a species belongs to [46]. Polychaetes may also have acquired a mechanism to systematically form spherocrystals during their evolutionary processes. In order to clarify how the lifestyle or lineage of polychaetes is related to the formation of spherocrystals, we plan to expand the research to the shallow water in a future study, increase the number of species to observe the digestive tract, and also expose the polychaetes to sulfide to clarify the relationship with sulfur regulation.

## Conclusions

Collectively, the present study characterized spherocrystals in the digestive tract of *Paralvinella* spp. and found that Polynoidae. gen. sp. regulated internal sulfur by different mechanisms, even though they lived on the same chimney. *Paralvinella* spp. may regulate sulfur by forming spherocrystals in the digestive tract. In contrast, Polynoidae. gen. sp. appears to regulate sulfur using the hypotaurine/thiotaurine system. These differences are not due to the bacteria in the digestive tract. Thus, the difference in sulfur regulation is likely due to their own physiological systems, and the choice of the system may be related to their lifestyles or lineage. Therefore, it is necessary to investigate the digestive tract epithelial cells extensively, including in shallow waters.

### Supplementary Information

**Additional file 1:** **Fig. S1.** *Paralvinella* spp. before dissection (A) and after abdominal incision (B). Externally exposed tufts were collected as “branchiae”, and the area enclosed by dotted lines in the abdominal cavity was collected as the “digestive tract”. Other parts were used for analysis as “body wall”. All tissues used in this study were included in the analyses. Therefore, this image was taken on another cruise and fixed in 70% ethanol for reference.

**Additional file 2:** **Fig. S2.** Polynoidae. gen. sp. before dissection (A) and after abdominal incision (B). The esophageal glands and their connecting tubular segments were collected collectively as “digestive tract”. Other parts were used for analysis as body walls. All tissues used in this study were included in the analyses. Therefore, this image was taken on another cruise and fixed in 70% ethanol for reference.

**Additional file 3.** The number of 16S rRNA read counts of the bacterial species in the digestive tracts of *Paralvinella * spp. (*N*=1) and Polynoidae. gen. sp. (*N*=1).

## Data Availability

All data are available in the main text and the supplementary imformation file. Further information and requests for data should be directed to and will be fulfilled by the corresponding author.
