# Supplementary material for: Difference in sulfur regulation mechanism between tube-dwelling and free-moving polychaetes sympatrically inhabiting deep-sea hydrothermal chimneys
Source: Zoological Lett. 2023 Oct 4;9:18. doi: 10.1186/s40851-023-00218-5 (PMC10548688; doi:10.1186/s40851-023-00218-5)

**Additional file 1**

Fig. S1. *Paralvinella* spp. before dissection (A) and after abdominal incision (B). Externally exposed tufts were collected as “branchiae”, and the area enclosed by dotted lines in the abdominal cavity was collected as the “digestive tract”. Other parts were used for analysis as “body wall”. All tissues used in this study were included in the analyses. Therefore, this image was taken on another cruise and fixed in 70% ethanol for reference.


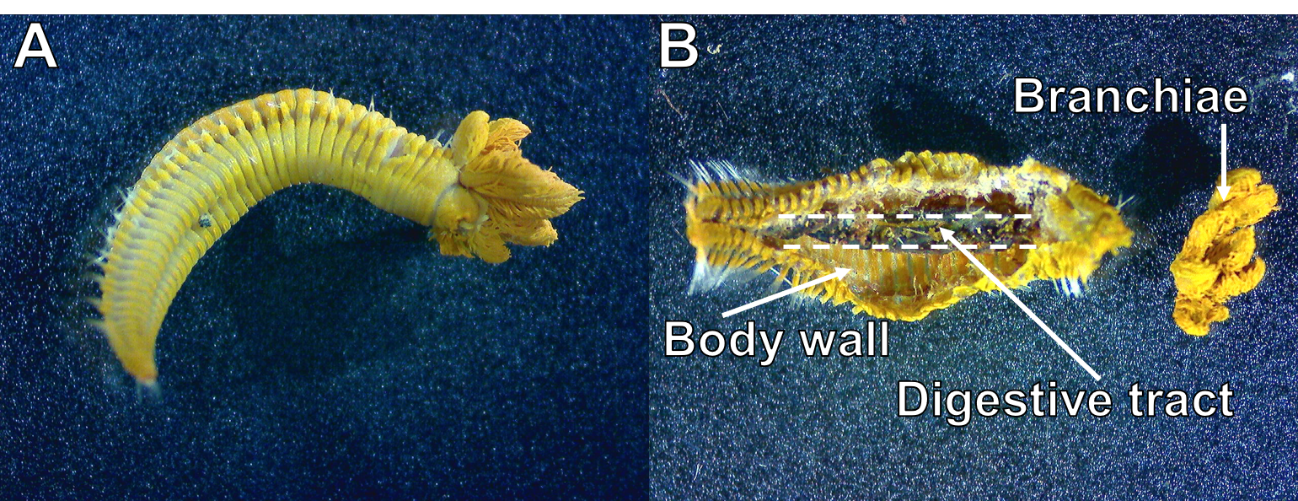

Supplement: Supplementary file 1 — Additional file 1: Fig. S1. Paralvinella spp. before dissection (A) and after abdominal incision (B). Externally exposed tufts were collected as “branchiae”, and the area enclosed by dotted lines in the abdominal cavity was collected as the “digestive tract”. Other parts were used for analysis as “body wall”. All tissues used in this study were included in the analyses. Therefore, this image was taken on another cruise and fixed in 70% ethanol for reference. [file 40851_2023_218_MOESM1_ESM.docx]
