# Supplementary material for: Difference in sulfur regulation mechanism between tube-dwelling and free-moving polychaetes sympatrically inhabiting deep-sea hydrothermal chimneys
Source: Zoological Lett. 2023 Oct 4;9:18. doi: 10.1186/s40851-023-00218-5 (PMC10548688; doi:10.1186/s40851-023-00218-5)
Supplement: Supplementary file 3 — Additional file 3. The number of 16S rRNA read counts of the bacterial species in the digestive tracts of Paralvinella spp. (N=1) and Polynoidae. gen. sp. (N=1). [file 40851_2023_218_MOESM3_ESM.docx]

**Additional file 3**

The number of 16S rRNA read counts of the bacterial species in the digestive tracts of *Paralvinella* spp. (*N*=1) and Polynoidae. gen. sp. (N=1)

| *Paralvinella* spp. | |  | Polynoidae. gen. sp. | |
| --- | --- | --- | --- | --- |
| Taxon Name | Taxon Count |  | Taxon Name | Taxon Count |
| *Variovorax boronicumulans* | 16758 |  | *Variovorax boronicumulans* | 23951 |
| *Variovorax paradoxus* | 8673 |  | *Variovorax paradoxus* | 12717 |
| *Variovorax guangxiensis* | 8620 |  | *Variovorax guangxiensis* | 12264 |
| *Xenophilus arseniciresistens* | 8256 |  | *Xenophilus arseniciresistens* | 11515 |
| *Acidovorax avenae* | 5005 |  | *Acidovorax citrulli* | 7498 |
| *Acidovorax citrulli* | 3466 |  | *Mesorhizobium australicum* | 6272 |
| *Paraburkholderia fungorum* | 3243 |  | *Paraburkholderia fungorum* | 5484 |
| *Mesorhizobium australicum* | 2873 |  | *Acidovorax avenae* | 4533 |
| *Mesorhizobium qingshengii* | 1312 |  | *Mesorhizobium shangrilense* | 2134 |
| *Mesorhizobium ciceri* | 1311 |  | *Mesorhizobium qingshengii* | 1950 |
| *Carbophilus carboxidus* | 1283 |  | *Mesorhizobium ciceri* | 1948 |
| *Aminobacter anthyllidis* | 1283 |  | *Paraburkholderia phenazinium* | 1397 |
| *Paraburkholderia phenazinium* | 812 |  | *Paraburkholderia terricola* | 1396 |
| *Paraburkholderia terricola* | 812 |  | *Nitratifractor salsuginis* | 961 |
| *Sphingomonas kyungheensis* | 365 |  | *Sphingomonas kyeonggiensis* | 447 |
| *Sulfurovum aggregans* | 290 |  | *Sphingomonas naasensis* | 377 |
| *Mesorhizobium loti* | 285 |  | *Sphingomonas kyungheensis* | 349 |
| *Mesorhizobium opportunistum* | 273 |  | *Sphingomonas leidyi* | 338 |
| *Sulfurovum* sp. NBC37-1 | 267 |  | *Mesorhizobium loti* | 288 |
| *Sulfurovum lithotrophicum* | 265 |  | *Mesorhizobium opportunistum* | 268 |
| *Mesorhizobium amorphae* | 204 |  | *Sphingomonas insulae* | 262 |
| *Sphingomonas roseiflava* | 189 |  | *Mesorhizobium amorphae* | 248 |
| *Sphingomonas aquatilis* | 189 |  | *Sphingomonas desiccabilis* | 241 |
| *Sphingomonas echinoides* | 164 |  | *Escherichia hermannii* | 234 |
| *Sphingomonas alpina* | 164 |  | *Escherichia vulneris* | 132 |
| *Escherichia hermannii* | 136 |  | *Variovorax soli* | 130 |
| *Mesorhizobium shangrilense* | 111 |  | *Trabulsiella guamensis* | 124 |
| *Escherichia vulneris* | 81 |  | *Desulfobulbus elongatus* | 122 |
| *Variovorax soli* | 74 |  | *Salmonella subterranea* | 120 |
| *Mesorhizobium huakuii* | 71 |  | *Pantoea vagans* | 105 |
| *Trabulsiella guamensis* | 71 |  | *Sphingomonas pituitosa* | 105 |
| *Salmonella subterranea* | 68 |  | *Sulfurovum aggregans* | 91 |
| *Labrys miyagiensis* | 60 |  | *Desulfobulbus rhabdoformis* | 84 |
| *Pantoea vagans* | 58 |  | *Desulfobulbus propionicus* | 71 |
| *Xenophilus aerolatus* | 36 |  | *Clostridium perfringens* | 67 |
| *Sphingomonas kyeonggiensis* | 35 |  | *Sphingomonas trueperi* | 67 |
| *Moraxella osloensis* | 33 |  | *Staphylococcus epidermidis* | 65 |
| *Labrys wisconsinensis* | 32 |  | *Sulfurovum sp. NBC37-1* | 54 |
| *Sphingomonas naasensis* | 31 |  | *Sphingomonas aquatilis* | 49 |
| *Propionibacterium acnes* | 31 |  | *Oceanithermus profundus* | 48 |
| *Sphingomonas pituitosa* | 31 |  | *Xenophilus aerolatus* | 46 |
| *Lutimonas halocynthiae* | 29 |  | *Sulfurovum lithotrophicum* | 44 |
| *Cocleimonas flava* | 24 |  | *Phyllobacterium myrsinacearum* | 43 |
| *Actinobacillus delphinicola* | 22 |  | *Sphingomonas roseiflava* | 42 |
| *Delftia tsuruhatensis* | 22 |  | *Sphingomonas echinoides* | 41 |
| *Lutimonas saemankumensis* | 21 |  | *Sphingomonas alpina* | 41 |
| *Lutimonas vermicola* | 21 |  | *Methylobacterium jeotgali* | 40 |
| *Brevundimonas vesicularis* | 18 |  | *Hydrogenimonas thermophila* | 39 |
| *Cetobacterium ceti* | 18 |  | *Bradyrhizobium ottawaense* | 35 |
| *Arcobacter cibarius* | 17 |  | *Methylobacterium hispanicum* | 34 |
| *Arcobacter cryaerophilus* | 17 |  | *Staphylococcus petrasii* | 33 |
| *Delftia acidovorans* | 16 |  | *Staphylococcus caprae* | 31 |
| *Kocuria rhizophila* | 16 |  | *Staphylococcus pasteuri* | 31 |
| *Limnohabitans planktonicus* | 15 |  | *Staphylococcus warneri* | 31 |
| *Delftia* sp. Cs1-4 | 15 |  | *Moraxella nonliquefaciens* | 30 |
| *Ralstonia pickettii* | 13 |  | *Ralstonia syzygii* | 29 |
| *Methylobacterium jeotgali* | 12 |  | *Delftia tsuruhatensis* | 28 |
| *Bradyrhizobium manausense* | 12 |  | *Bradyrhizobium ingae* | 26 |
| *Sphingomonas insulae* | 12 |  | *Methylobacterium phyllostachyos* | 26 |
| *Staphylococcus epidermidis* | 12 |  | *Ralstonia insidiosa* | 25 |
| *Bradyrhizobium ganzhouense* | 11 |  | *Bradyrhizobium neotropicale* | 25 |
| *Gallibacterium salpingitidis* | 11 |  | *Psychrobacter adeliensis* | 25 |
| *Bradyrhizobium rifense* | 11 |  | *Bradyrhizobium lupini* | 25 |
| *Vespertiliibacter pulmonis* | 11 |  | *Moraxella catarrhalis* | 24 |
| *Delftia lacustris* | 11 |  | *Oceanithermus desulfurans* | 24 |
| *Bradyrhizobium icense* | 10 |  | *Methylobacterium tardum* | 24 |
| *Bradyrhizobium paxllaeri* | 10 |  | *Methylobacterium longum* | 24 |
| *Bradyrhizobium lupini* | 10 |  | *Methylobacterium oxalidis* | 23 |
| *Kocuria salsicia* | 9 |  | *Bradyrhizobium paxllaeri* | 23 |
| *Phyllobacterium myrsinacearum* | 9 |  | *Phyllobacterium loti* | 23 |
| *Sphingomonas dokdonensis* | 9 |  | *Methylobacterium trifolii* | 23 |
| *Actinobacillus scotiae* | 9 |  | *Bradyrhizobium icense* | 23 |
| *Rhodococcus rhodochrous* | 8 |  | *Sulfurimonas autotrophica* | 22 |
| *Brevundimonas nasdae* | 8 |  | *Mesorhizobium huakuii* | 22 |
| *Methylobacterium trifolii* | 7 |  | *Methylobacterium gregans* | 17 |
| *Pseudopelagicola gijangensis* | 7 |  | *Methylobacterium aerolatum* | 16 |
| *Actibacter sediminis* | 7 |  | *Phyllobacterium endophyticum* | 14 |
| *Loktanella litorea* | 7 |  | *Labrys miyagiensis* | 14 |
| *Jannaschia faecimaris* | 7 |  | *Brevundimonas vesicularis* | 12 |
| *Methylobacterium oxalidis* | 7 |  | *Propionibacterium acnes* | 12 |
| *Phyllobacterium loti* | 7 |  | *Sphingomonas dokdonensis* | 12 |
| *Confluentimicrobium lipolyticum* | 6 |  | *Labrys wisconsinensis* | 10 |
| *Staphylococcus petrasii* | 6 |  | *Delftia acidovorans* | 9 |
| *Staphylococcus caprae* | 6 |  | [*Eubacterium*] *tenue* | 9 |
| *Phyllobacterium endophyticum* | 6 |  | *Delftia* sp. Cs1-4 | 9 |
| *Rhodovulum kholense* | 6 |  | *Paraburkholderia phenoliruptrix* | 8 |
| *Phreatobacter oligotrophus* | 6 |  | *Pseudomonas tolaasii* | 8 |
| *Ahrensia kielensis* | 6 |  | *Photobacterium phosphoreum* | 8 |
| *Peptoclostridium difficile* | 6 |  | *Phyllobacterium brassicacearum* | 8 |
| *Microbacterium kyungheense* | 6 |  | *Delftia lacustris* | 8 |
| *Methylobacterium phyllostachyos* | 6 |  | *Ralstonia pickettii* | 8 |
| [*Eubacterium*] *tenue* | 5 |  | *Ralstonia solanacearum* | 7 |
| *Staphylococcus pasteuri* | 5 |  | *Psychrobacter maritimus* | 6 |
| *Pelomonas saccharophila* | 5 |  | *Brevundimonas nasdae* | 6 |
| *Roseovarius mucosus* | 5 |  | [*Clostridium*] *sordellii* | 6 |
| *Hydrogenophaga carboriunda* | 5 |  | *Photobacterium piscicola* | 6 |
| *Staphylococcus chromogenes* | 5 |  | *Psychrobacter aquaticus* | 6 |
| *Donghicola eburneus* | 5 |  | *Roseibacterium elongatum* | 6 |
| *Poseidonocella sedimentorum* | 5 |  | *Dermacoccus profundi* | 6 |
| *Tepidimonas taiwanensis* | 5 |  | *Psychrobacter piscatorii* | 5 |
| *Tepidimonas ignava* | 5 |  | *Actinobacillus scotiae* | 5 |
| *Methylobacterium aerolatum* | 5 |  | *Psychrobacter ciconiae* | 5 |
| *Loktanella koreensis* | 5 |  | *Psychrobacter cryohalolentis* | 5 |
| *Aquamicrobium aerolatum* | 4 |  | *Tepidimonas taiwanensis* | 5 |
| *Roseovarius aestuarii* | 4 |  | *Paeniglutamicibacter antarcticus* | 4 |
| *Sulfitobacter litoralis* | 4 |  | *Achromobacter animicus* | 4 |
| *Polaribacter marinivivus* | 4 |  | *Dermacoccus abyssi* | 4 |
| [*Clostridium*] *sordellii* | 4 |  | *Pseudomonas poae* | 4 |
| *Microbacterium aerolatum* | 4 |  | *Pseudomonas mucidolens* | 4 |
| *Tomitella biformata* | 4 |  | *Pseudomonas straminea* | 4 |
| *Tenacibaculum caenipelagi* | 4 |  | *Aliivibrio fischeri* | 4 |
| *Paraburkholderia phenoliruptrix* | 4 |  | *Hydrogenophaga carboriunda* | 4 |
| *Loktanella maritima* | 4 |  | *Tepidimonas ignava* | 4 |
| *Asaccharospora irregularis* | 4 |  | *Pseudomonas punonensis* | 4 |
| [*Clostridium*] *ghonii* | 4 |  | *Kosakonia pseudosacchari* | 3 |
| *Polaribacter huanghezhanensis* | 4 |  | *Achromobacter xylosoxidans* | 3 |
| *Vibrio cyclitrophicus* | 4 |  | *Litoreibacter halocynthiae* | 3 |
| *Microbacterium marinilacus* | 4 |  | [*Clostridium*] *ghonii* | 3 |
| *Polaribacter porphyrae* | 4 |  | *Aminobacter niigataensis* | 3 |
| *Thalassobius maritimus* | 4 |  | *Psychrobacter salsus* | 3 |
| *Octadecabacter arcticus* | 3 |  | *Tateyamaria omphalii* | 3 |
| *Pseudaminobacter defluvii* | 3 |  | *Litoreibacter ponti* | 3 |
| *Vibrio chagasii* | 3 |  | *Halomonas axialensis* | 3 |
| *Jannaschia* sp. CCS1 | 3 |  | *Litoreibacter ascidiaceicola* | 3 |
| *Litoreibacter arenae* | 3 |  | *Bauldia consociata* | 3 |
| *Methylobacterium tardum* | 3 |  | *Acidovorax wautersii* | 3 |
| *Lentibacter algarum* | 3 |  | *Methylobacterium extorquens* | 3 |
| *Methylobacterium longum* | 3 |  | *Shewanella algidipiscicola* | 3 |
| *Loktanella ponticola* | 3 |  | *Shewanella marina* | 3 |
| *Avibacterium endocarditidis* | 3 |  | *Aliivibrio sifiae* | 3 |
| *Kocuria varians* | 3 |  | *Photobacterium iliopiscarium* | 3 |
| *Vibrio toranzoniae* | 3 |  | *Shewanella violacea* | 3 |
| *Aliiroseovarius sediminilitoris* | 3 |  | *Shewanella algae* | 3 |
| *Aminobacter niigataensis* | 3 |  | *Dermacoccus barathri* | 3 |
| *Aliiroseovarius crassostreae* | 3 |  | *Psychrobacter pulmonis* | 2 |
| *Vibrio gallaecicus* | 3 |  | *Sphingomonas changbaiensis* | 2 |
| *Litoreibacter ponti* | 3 |  | *Ketogulonicigenium vulgare* | 2 |
| *Giesbergeria sinuosa* | 2 |  | *Halomonas zhaodongensis* | 1 |
| *Ramlibacter ginsenosidimutans* | 2 |  | *Aquamicrobium aerolatum* | 1 |
| *Aquamicrobium terrae* | 2 |  | *Acidovorax delafieldii* | 1 |
| *Curvibacter delicatus* | 1 |  | *Psychrobacter jeotgali* | 1 |
| *Pandoraea thiooxydans* | 1 |  | *Pseudaminobacter defluvii* | 1 |
| *Methylobacterium radiotolerans* | 1 |  | *Rubrivivax gelatinosus* | 1 |
| *Mesorhizobium plurifarium* | 1 |  | *Psychrobacter urativorans* | 1 |
| *Sphingomonas trueperi* | 1 |  | *Acidovorax ebreus* | 1 |
| *Giesbergeria anulus* | 1 |  | *Tomitella biformata* | 1 |
| *Shewanella marina* | 1 |  | *Psychrobacter immobilis* | 1 |
| *Aquincola tertiaricarbonis* | 1 |  | *Oceanibacterium hippocampi* | 1 |
| *Paraburkholderia glathei* | 1 |  | *Vogesella oryzae* | 1 |
| *Acinetobacter seifertii* | 1 |  | *Flavobacterium ummariense* | 1 |
| *Aliivibrio fischeri* | 1 |  | *Staphylococcus pettenkoferi* | 1 |
| *Limnohabitans australis* | 1 |  | *Mesorhizobium septentrionale* | 1 |
| *Achromobacter animicus* | 1 |  | *Defluviicoccus vanus* | 1 |
| *Achromobacter xylosoxidans* | 1 |  | *Brevundimonas aurantiaca* | 1 |
| *Paracoccus koreensis* | 1 |  | *Rhodovulum mangrovi* | 1 |
| *Paraburkholderia caryophylli* | 1 |  | *Brucella papionis* | 1 |
| *Polaromonas naphthalenivorans* | 1 |  | *Ralstonia pseudosolanacearum* | 1 |
| *Comamonas composti* | 1 |  | *Comamonas testosteroni* | 1 |
| *Litoreibacter halocynthiae* | 1 |  | *Sphingomonas koreensis* | 1 |
| *Polaribacter butkevichii* | 1 |  | *Altererythrobacter xinjiangensis* | 1 |
| *Psychrobacter arcticus* | 1 |  | *Paraburkholderia sordidicola* | 1 |
| *Vitellibacter vladivostokensis* | 1 |  | *Achromobacter spanius* | 1 |
| *Hydrogenophaga pseudoflava* | 1 |  | *Afipia massiliensis* | 1 |
| *Salipiger mucosus* | 1 |  | *Paracoccus kocurii* | 1 |
| *Hydrogenophaga defluvii* | 1 |  | *Diaphorobacter oryzae* | 1 |
| Unclassified hit | 3486 |  | *Pseudarthrobacter phenanthrenivorans* | 1 |
| total | 71595 |  | *Burkholderia multivorans* | 1 |
|  |  |  | *Methylacidiphilum infernorum* | 1 |
|  |  |  | *Sulfitobacter dubius* | 1 |
|  |  |  | *Celeribacter indicus* | 1 |
|  |  |  | *Halomonas olivaria* | 1 |
|  |  |  | *Sphingomonas adhaesiva* | 1 |
|  |  |  | *Methylobacterium radiotolerans* | 1 |
|  |  |  | *Curtobacterium plantarum* | 1 |
|  |  |  | *Acinetobacter johnsonii* | 1 |
|  |  |  | *Halomonas aquamarina* | 1 |
|  |  |  | *Chryseobacterium yonginense* | 1 |
|  |  |  | *Rhodoferax antarcticus* | 1 |
|  |  |  | *Diaphorobacter aerolatus* | 1 |
|  |  |  | *Subsaxibacter broadyi* | 1 |
|  |  |  | *Paraburkholderia glathei* | 1 |
|  |  |  | *Altererythrobacter marinus* | 1 |
|  |  |  | *Actinobacillus delphinicola* | 1 |
|  |  |  | *Sphingomonas canadensis* | 1 |
|  |  |  | *Burkholderia jiangsuensis* | 1 |
|  |  |  | *Candidatus Moranella endobia* | 1 |
|  |  |  | *Comamonas composti* | 1 |
|  |  |  | *Spirillospora albida* | 1 |
|  |  |  | *Cribrihabitans marinus* | 1 |
|  |  |  | *Psychrobacter fozii* | 1 |
|  |  |  | *Luteolibacter cuticulihirudinis* | 1 |
|  |  |  | *Psychrobacter submarinus* | 1 |
|  |  |  | *Bizionia paragorgiae* | 1 |
|  |  |  | *Chryseobacterium carnis* | 1 |
|  |  |  | *Aliivibrio wodanis* | 1 |
|  |  |  | *Acidovorax radicis* | 1 |
|  |  |  | *Comamonas serinivorans* | 1 |
|  |  |  | Unclassified hit | 20899 |
|  |  |  | total | 120852 |
